# Seroprevalence study of peste des petits ruminants in sheep and goats in the northern region of India

**DOI:** 10.14202/vetworld.2020.1573-1580

**Published:** 2020-08-13

**Authors:** Vinayagamurthy Balamurugan, Bibitha Varghese, Kirubakaran Vinod Kumar, Dhanavelu Muthuchelvan, R. Dheeraj, Gurrappanaidu Govindaraj, Kuralayanapalya Puttahonnappa Suresh, Divakar Hemadri, Parimal Roy

**Affiliations:** 1 Indian Council of Agricultural Research - National Institute of Veterinary Epidemiology and Disease Informatics, Bengaluru, Karnataka, India; 2 Division of Virology, ICAR - Indian Veterinary Research Institute, Nainital, Uttarakhand, India

**Keywords:** cross-sectional study, India, northern region, peste des petits ruminants, seroprevalence, sheep and goats

## Abstract

**Background and Aim:**

Peste des petits ruminants (PPR) is a contagious, World Organization for Animal Health notifiable, economically important, transboundary morbilliviral disease of sheep and goats. Studying seroprevalence of PPR from different geographical areas under varying agro-climatic conditions may help in formulating effective and appropriate disease control strategies under the ongoing national PPR control program. The present cross-sectional study describes the prevalence of PPR virus antibodies in sheep and goats in the various epidemiological units in different states (Haryana, Himachal Pradesh [HP], Jammu and Kashmir [J&K], Punjab, Uttarakhand [UK], and Uttar Pradesh [UP]) of the northern region of India.

**Materials and Methods:**

A total of 5843 serum samples (sheep [n=2463] and goats [n=3380]) were collected by stratified random sampling method from 322 epidemiological units in the studied region during 2017-2018 and tested for PPR virus (PPRV) antibodies by competitive ELISA.

**Results:**

The results revealed that an overall seroprevalence of 44.05% (2574/5843) with 57.32%, 55.22%, 65.69%, 37.09%, 32.73%, and 29.35% prevalence of PPRV antibodies in small ruminants in Haryana, Punjab, UP, HP, J&K, and UK states, respectively. Further, Chi-squared test revealed an association of PPRV antibodies in goats (χ^2^=252.28, p<0.01) and sheep (χ^2^=192.12, p<0.01) across different states in the region.

**Conclusion:**

The seroprevalence in majority of the epidemiological units (n=130) in sheep and goats in the studied region had <30%. This necessitates comprehensive, rigorous, continuous vaccination and active surveillance programs for few more years to achieve the desired 70% seroprevalence level of PPRV antibodies in population and to make the northern region of India, as PPR free zone.

## Introduction

Peste des petits ruminants (PPR), otherwise known as “Plague of Small Ruminants” or “Goat Plague,” is an acute, highly contagious, and economically important and World Organization for Animal Health (OIE) notifiable transboundary viral disease of sheep and goats. PPR is caused by the small ruminants *Morbillivirus* (formerly known as PPR virus [PPRV]), a member of the genus *Morbillivirus* of the family *Paramyxoviridae* (http://ictvonline.org/virusTaxonomy.asp). The disease is clinically characterized by high fever (pyrexia), oculonasal discharges, oral necrotizing and erosive ulcers, stomatitis, gastroenteritis, diarrhea, and bronchopneumonia [1]. The disease due to its transboundary nature causes major constraints in improving the productivity of small ruminants in enzootic countries, and it causes huge economic losses, and it significantly influences the livestock sector economy [2]. Because of the vast socio-economic impacts of PPR, the global scientific community stressed the requirement to eradicate PPR, with the adoption of the PPR global control and eradication strategy (GCES) by 2030 [3]. In this direction, the Food and Agriculture Organization and OIE jointly initiated the strategic plan, PPR-Global Eradication Program, for the control and eradication of PPR. This program has been launched for the initial period of 2017-2021 and put into action with the adoption of PPR GCES.

In India, sheep and goats play an essential role in the socio-economic development of rural households and are generally referred for “Any Time Money” to rural landless, marginal, and small landholding farmers. These animals mostly constitute an important productive asset and generate a flow of income and employment for their livelihood. PPR is enzootic, and several outbreaks occur regularly in different parts of India [1], and it causes significant economic losses [2] in terms of morbidity, mortality, and productivity losses with trade limitation [1,4]. Some of the states in India practiced focused vaccination (vaccination limited to the place of the outbreak with a radius of 3-10 km to contains the disease spread) in the outbreaks situation for the control of the PPR since 2002 [5]. However, the strategic mass vaccination program (vaccination covering the entire small ruminants population above the age of 4 months old and subsequent biannual/annual vaccination of naïve young population and unvaccinated animals) was implemented in some of the states [6] through the national control program on PPR (PPR-CP) since 2010 and 2014 (http://www.dahd.nic.in) for the control and eradication of the disease even before the global framework was planned [7].

Nevertheless, neither a surveillance plan nor systematic post-vaccination monitoring and/or evaluation was initiated to assess the effectiveness of the vaccination and its strategies. Several PPR outbreaks go unrecorded due to under-reporting or non-reporting due to poor surveillance system in India. Further, outbreaks are being reported regularly in some of the states despite focus vaccination and a few sporadic outbreaks were reported in mass vaccination program implemented states or geographically restricted areas of hilly terrain in the states of Uttarakhand (UK) and Himachal Pradesh (HP) and Jammu and Kashmir (J&K). Nevertheless, systematic epidemiological surveys for the state or region or zone have not been conducted except for a few studies [8-11]. Moreover, studying the prevalence and generating evidence of the level of PPRV antibodies in the target population is paramount importance to formulate and implement a proper strategic disease control vaccination program in a particular geographical area with a long-term plan to eradicate PPR by 2030.

Therefore, the present cross-sectional serosurveillance, being employed to establish the prevalence of PPRV antibodies level at epidemiological units (epi-units) in the target sheep and goats population at disaggregated levels (states) in the study region at a given period from 2017 to 2018.

## Materials and Methods

### Ethical approval

The manuscript does not contain animal experimental trials. No ethical clearance is required for collecting small volumes of blood samples required for seroepidemiological studies, as per Committee for the Purpose of Control and Supervision of Experiments on Animals guidelines. Moreover, the samples were collected by well-trained veterinarians considering animal welfare regulations.

### Study region

The northern zonal region of India representing both a geographic and political or zonal administrative section of the country, and it comprises the states of Haryana, HP, J&K, Punjab, Uttar Pradesh (UP), and UK, and the Union Territories (UTs) of Delhi and Chandigarh. In this region, PPR outbreaks in some states have been reported continuously since 1996 [4,12-16], and many states have not implemented the PPR-CP, though directed to adopt since 2014-2015 (http://www.dahd.nic.in/) and the outbreaks are being reported substantially. Delhi, and Chandigarh UTs were excluded in this study due to the very small geographical area, meager animal population, as well as no PPR outbreaks were reported (http://www.dahd.nic.in/).

### Study period and sampling

As a part of monitoring the status of livestock diseases, Indian Council of Agricultural Research-National Institute of Veterinary Epidemiology and Disease Informatics (ICAR-NIVEDI) carried out a cross-sectional seroprevalence study between June 2017 and March 2018 to ascertain the prevalence status of PPRV antibodies in small ruminants’ population in the various epi-units in different states of the studied region. The sample size was determined for the finite or large population as per Cochran [17] formula N=Z^2^ [p (1−p/e^2^] using the epitool, where N=sample size, Z=95% confidence level, p=30% proportion {(animal unit-level prevalence of 30% was considered as per GCES [18] and the seroprevalence of PPR in India before the implementation of vaccination [4]}, e is the precision level (5%). Based on these inputs, a total sample size of 323 was determined (http://epitools.ausvet.com.au/content.php?page=1Proportion), for the target populations in the study region. However, after considering the attrition rate of 10%, the total arrived sample size was 356. In the Indian context, the village is distinct and considered as epi-unit in the studied states, as described earlier [10]. The list of villages in each state having more than 200 small ruminants (with inclusion and exclusion criteria as per the 19^th^ Livestock Census, 2012) population (http://www.dahd.nic.in/) was shortlisted, which accounted for the sampling frame. The multistage stratified random sampling method was adopted for collecting serum samples from different states in the studied region.

In the first stage, the states were stratified, and the estimated 60 sampling primary epi-units (villages) were allocated randomly to the different districts in each stratum (state) using R software [19]. Randomization of villages was done based on the in-house NIVEDI developed software epi-calculator. In the next stage, in each of the selected villages, the number of secondary animal unit samples within an epi-unit was calculated by the hypergeometric distribution as per GCES guidelines [18], and a maximum of 11 samples to be collected was determined by epi-calculator (https://www.nivedi.res.in/Nadres_v2/Epical/stratified/random_sampling.php). Therefore, a maximum sample of 1320 animal units (660 for each of the target [sheep or goats] species) to be sampled from each state has arrived for the present serosurvey. In the third stage, the flocks/households to be surveyed in each of the selected villages were randomly selected. In the epi-unit, where only either sheep or goats reared, a maximum of 11 samples of either species was collected based on the available target population.

### Samples and screening

**Figure-1:**
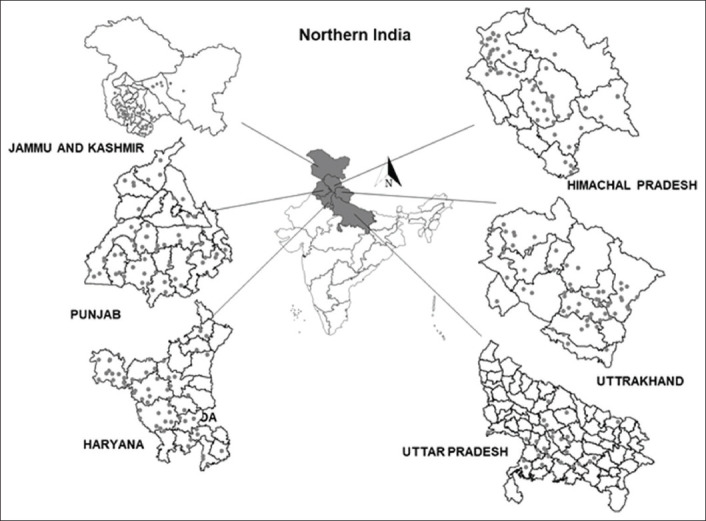
The surveyed epi-units (villages) location is depicted (as ■ a dot) in the GIS Map of the studied states in the northern region of India.

In each epi-unit, serum samples were collected randomly from selected flocks/households as per the sampling plan through All India Co-ordinated Research Project on Animal Disease Monitoring and Surveillance (AICRP on ADMAS), a collaborating center of ICAR-NIVEDI, in the respective states. The surveyed epi-units in the states of the studied northern region are depicted in GIS Map (Figure-1) using QGIS Software 2.18.6 version (QGIS team, Switzerland). The collected sera were labeled and transported in an ice-cool shipment container to the laboratory, and the samples were stored at −20°C until further use. All the sera were tested by competitive ELISA, which is being employed for the serosurveillance or seromonitoring of PPR in India for the detection of PPRV specific antibodies [20], which were measured in terms of percentage inhibition (PI) according to Singh *et al*. [20] protocol and samples with a PI of ≥40% were considered as a positive.

### Statistical analysis

The seroprevalence was estimated by the number of positive versus numbers of tested samples, as per the described method [21]. The Chi-squared test was carried out in MS-office Excel 2016, as per the described method [22] to understand the association (the null hypothesis [H_0_] is independent) in the presence of PPRV antibodies in sheep and goats across the states and districts, and between the species in the northern region. The working hypothesis of the homogeneous occurrence of PPRV antibodies in the target populations in the epi-units in different states of the study region was considered. Further, the annual growth rate (GR) of PPRV antibodies if mass vaccination continues regularly for the different states was assessed, to predict the number of years of vaccination required to achieve 70% prevalence [3] using mathematical formula {GR= ([(b−a)/a] × 100)/N} as described earlier [8], where a-Base level prevalence (30%), b-Study year prevalence, and N-No. of years (3 years). The number of years considered for growth assessment was three, due to the vaccinated populations would have turned over by then as sheep and goat’s typical lifespan is 3 years. Further, keeping in view the turnover of the populations, the calculated growth was discounted by 30% each year [4].

## Results

**Figure-2:**
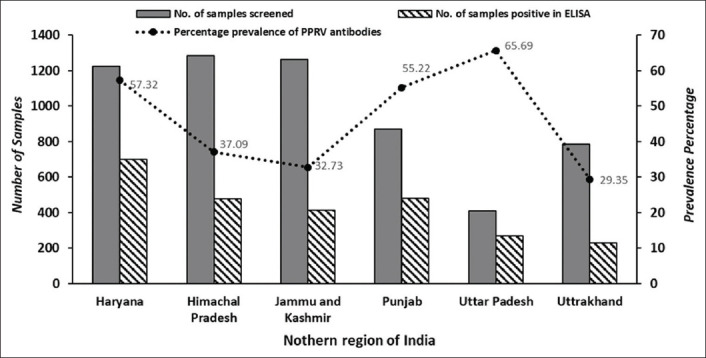
State-wise seroprevalence of peste des petits ruminants in small ruminants in the northern region of India.

**Figure-3:**
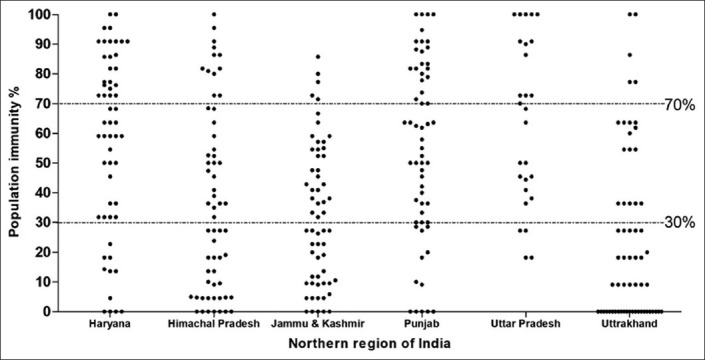
Distribution of epi-units based on percent positivity levels of peste des petits ruminants virus antibodies in the studied region.

The observed prevalence of PPRV antibodies in the small ruminants was 57.32%, 55.22%, 65.69%, 37.09%, 32.73%, and 29.35% in Haryana, Punjab, and UP, HP, J&K, and UK states, respectively, with an overall seroprevalence of 44.05% (2574/5843) in the studied states of the northern region. State-wise details of sera screened, and their percent positivity with seroprevalence are presented in Table-1 and Figure-2, and the percentage prevalence of PPRV antibodies in various epi-units of different states is depicted in Figure-3. In majority of the epidemiological units (n=130), seroprevalence was <30%, with only 79 epi-units had >70% in the studied region. The results of the Chi-squared test revealed that there exists an association of PPRV antibodies in goats (χ^2^=252.28, p<0.01) and sheep (χ^2^=192.12, p<0.01) across different states in the region, as most of the states practiced PPR vaccination. Further, the analysis also indicated the association of PPRV antibodies in sheep (χ^2^=48.43, p<0.01) and goats (χ^2^=44.30, p<0.01) across different districts of HP. The district-wise details of seroprevalence of PPR in small ruminants in Haryana, Punjab, UP, HP, J&K, and UK states are available from the authors as supplementary tables on request. Further, the calculated annual GR for accomplishing the desired percentage of antibodies prevalence in different states is shown in Table-1.

**Table-1:** State-wise details of the small ruminant samples tested and its results of PPRV antibodies prevalence and calculated annual growth rate in the northern region of India.

Name of the States	Number of Tehsil/Block	Numer of Village/Epi-Unit	Number of samples screened			Number of samples positive			Seroprevalence % at Epi-Unit level (number)			Prevalence of PPRV antibodies (CI-value at 95%)			Calculated annual growth rate for accomplishing the desired percentage of antibodies prevalence^$^			
				
			Sheep	Goats	Total	Sheep	Goats	Total	<30	30-70	>70	Sheep	Goats	Total	2017-2018 in the present study	2018-2019	2019-2020	2020-2021
Haryana	31	56	610	613	1223	351	350	701	11	21	24	57.54 (54-61)	57.1 (53-61)	57.32 (55-60)	57.32	61.70	83.12	104.54
Himachal Pradesh	37	60	646	640	1286	215	262	477	29	19	12	33.28 (30-37)	40.94 (37-45)	37.09 (32-37)	-	-	-	-
Jammu and Kashmir	34	60	632	633	1265	219	195	414	30	25	5	34.65 (31-38)	30.81 (27-35)	32.73 (30-35)	-	-	-	-
Punjab	40	59	312	559	871	179	302	481	15	23	21	57.37 (52-63)	54.03 (50-58)	55.22 (52-58)	55.22	61.45	82.73	104.02
Uttar Pradesh	26	27	131	280	411	104	166	270	4	11	12	79.39 (72-85)	59.29 (53-65)	65.69 (61-70)	65.69	93.99	132.40	-
Uttarakhand	42	60	132	655	787	79	152	231	41	14	5	59.85 (51-68)	23.21 (20-27)	29.35 (26-33)	-	-	-	-
Total	210	322	2463	3380	5843	1147	1427	2574	130	113	79	46.57 (45-49)	41.22 (41-44)	44.05 (43-45)	-	-	-	**-**
	Chi-square value (sheep-χ^2^=192.12, p<0.01; goats-χ^2^=252.28, p<0.01) and (species χ^2^=192.12, p<0.01)																	

$ For Himachal Pradesh, Jammu and Kashmir, and Uttarakhand state Annual growth rate could not be carried out due to the prevalence of PPRV antibodies during 2017-2018 was near to the 30% base level, which is the base level seroprevalence employed for the calculation. PPRV=Peste des petits ruminants virus, CI=Confidence interval

## Discussion

As per the 20^th^ Livestock Census, 2019, India has 148.88 million goats and 74.26 million sheep (http://dahd.nic.in/division/provisional-key-results-20th-livestock-census accessed on 25^th^ October 2019), and the population has increased by 10.14% goat and 14.13% sheep when compared to the 19^th^ Livestock Census, 2012. For the effective control and eradication of PPR, disease reporting, epidemiology, surveillance and monitoring of disease, support of diagnostics, and vaccination of the susceptible populations are highly imperative [1]. Information on the seroprevalence of PPR in different domestic and wildlife ruminant species has been reported from different enzootic countries in Africa, the middle east, and Asia [4,9,11,13,15,23-29]. The prevalence of PPRV antibodies in sheep and goats indicates either the subclinical or in-apparent suspected infection or naturally infected and recovered animals and has specific implications in epidemiological perspectives since it highlights the prevalence under natural non-vaccination situation or it may indicate animal’s immune response to the vaccine [29], as the prevalence of antibodies in adult animals is not always indicative of infection, as there is always a high probability of these animals receiving vaccination once during a lifetime. Earlier studies conducted at the various period since 1996, generated the baseline data on the PPRV antibodies in small ruminants in some states of the northern region of India [4,13,15,30-32]. Nevertheless, the majority of the reports indicated only the regional isolated data using a limited number of samples [4,30-32]. Hence, the present systematic cross-sectional survey undertaken during 2017-2018, assessed the prevalence status of PPRV antibodies in sheep and goats at the epi-unit levels of the states (disaggregated levels) in North India.

Haryana state initiated the focused-vaccination since 2011-2012, and controlled the reported outbreaks [13] and reduced the epidemic level of PPR (http://krishikosh.egranth.ac.in/handle/1/5810028108). The observed overall prevalence of PPRV antibodies was 57.32%, with the seroprevalence of 57.54% (351/610) in sheep and 57.1% (350/613) in goats. However, only 24 out of 56 epi-units tested covering 31 blocks in 15 districts, had >70% prevalence level of antibodies, which implies uniform vaccination in all the villages in the different districts of the state, is not being practiced. Similarly, Punjab state adopted mass vaccination during 2014-2015 for the control of the outbreaks [14]. The observed prevalence of antibodies was 55.22%, with the seroprevalence of 57.37% (179/312) in sheep and 54.03% (302/559) in goats, which could be due to the mass vaccination of small ruminants. However, only 21 out of 59 epi-units tested covering 40 blocks in 17 districts of the state had >70% prevalence level of antibodies, which implies vaccination is not being practiced in all the epi-units of the districts in the state. Whereas, UP state initiated focused vaccination in the area of the outbreaks since 2006-2007, as and when required, and adopted mass vaccination during 2011 under PPR-CP with an overall poor coverage of 0.83%-22.68% in 2017-2018 (except 2015-2016 during which the coverage was 86.35%). Moreover, only 12 out of 27 epi-units tested covering 26 blocks in 17 districts, had >70% prevalence level of PPRV antibodies. It indicates mass vaccination may not be practiced in all the districts in UP as per the stipulated strategic plan [12,33], even though, tested samples showed an overall seroprevalence of 79.39% (104/131) in sheep and 59.29% (166/280) in goats.

HP state has initiated focused vaccination in the area of the outbreaks since 2003-2004, as and when required to contain the epidemic and controlled the outbreaks. As per record, the state implemented national PPR-CP and adopted mass vaccination during 2014-2015 with an overall coverage of 35% in 2014-2015; 42% in 2015-2016; and 39% in 2017-2018 with an average coverage of only 35-40%, which indicates regular mass vaccination program as per PPR-CP strategy, is not being practiced in HP. Further, only 12 out of 60 epi-units tested covering 37 blocks in eight districts, had >70% prevalence level of PPRV antibodies, with an overall seroprevalence of 33.28% (215/646) in sheep and 40.94% (262/640) in goats. In J&K state, so far, quite a number of the outbreaks have been reported and fairly prevalent in nomadic sheep and goats with the evidence of natural transmission [30,34], as the state has not adopted vaccination program. The observed overall base level of 32.58% (34.65% [219/632] in sheep and 30.81% [195/633] in goats) seroprevalence might be due to the non-adoption of the mass vaccination. Moreover, only five out of 60 epi-units covering the 34 blocks in 14 districts had >70 prevalence levels of PPRV antibodies. A few positive samples could be due to the earlier vaccinated or recovered infected animals introduced from the other parts of India, as stated earlier by most of the researchers. Similarly, the UK followed focused vaccination since 2011-2012, to contain the outbreaks, but the state had not adopted a mass vaccination campaign in line with the PPR-CP since 2014 like other studied states in North India.

The observed low seroprevalence of PPR in these (UK, HP, and J&K) states might be because the samples were randomly collected from apparently healthy animals and not from PPR suspected animals. Furthermore, the regional difference in the prevalence of the PPRV antibodies based on the relative population has also been reported [4,11]. In general, the low seroprevalence of PPR in sheep and goats in an ecological and geographically niche of hilly terrains could be because the topology of the region *per se* restricts migration of animals from the nearby region, low population density, availability of low grazing area, and thus close contact of animals are avoided. Besides, different studies demonstrate the various percentage of seroprevalence in apparently healthy animals from different states of India at various serological surveys. The seroprevalence of 34.52% in goats and 54.62% in sheep was reported in Haryana and Delhi [15], whereas seroprevalence of 29.16% in sheep and 28.70% in goats reported in Jammu [30]. Whereas Bhanuprakash *et al*. [31] reported the prevalence of 41.7% in sheep while studying the status of PPR, sheeppox, and bluetongue virus antibodies in the northern states of India. All these studies have generally indicated the variation in the PPR outbreaks and associated risk factors such as species, age, sex and husbandry practices, and transboundary migration of animals from neighboring or border states.

The variation in seroprevalence could also be attributed to sampling size variation across studies, prevailing management practices, humidity, or season as reported earlier [4]. However, the present study was systematic with appropriate sampling plan, procedure, and design with a specified level of confidence intervals and desired precision with the maximum statistical sample size for the finite large population representing the target populations from different epidemiological units of the studied region. Nevertheless, the present study needs to be visualized with certain limitations, like the disease-associated risk/host factors such as breed, sex, age, etc.were not available for further multi-factorial regression analysis. In some states, the target epi-units, as per the sampling plan, could not be surveyed due to administrative constraints.

Further, the number of years vaccination need to be continued to reach the desired 70% prevalence levels of PPRV antibodies [3,18] in sheep and goats in all the epi-units in different states of the studied region was determined (Table-1), based on the annual GR of the prevalence of antibodies. Moreover, for the control of the disease, Fournié *et al*. [35] recently stated that viral spread could be prevented (controlled) if the proportion of immune small ruminant is kept permanently above 37% in at least 71% of village population in an endemic setting by fitting a meta-population simulating the model. However, due to the high turnover of these small ruminants population, maintaining the fraction of immune animals above this threshold would require high vaccine coverage within villages/epi-units. In the present study, this estimates corresponded with the observed 37% prevalence of antibodies in 70% of the tested epi-units, in Punjab and Haryana states, which might be prevented or restricted the spread of infection, as there were no outbreaks, have been reported from these states for the past couple of years.

Whereas the HP, UK, and J&K states had the only seroprevalence of 30-35%, and these states need to vaccinate the small ruminants for 4-5 years (2023-2024) to achieve desired 70% levels of PPRV antibodies as envisaged in PPR-CP. Therefore, for the control and eradication of PPR, 80-90% coverage of vaccination of the risk population is required to achieve the desirable herd immunity to prevent the active transmission of the disease by considering the other epidemiological factors [36]. For that, vaccination covering the entire population initially, subsequently bi-annual vaccination covering the naïve young population need to be adopted, as per PPR control and eradication strategic plan.

## Conclusion

The present survey provides information on the seroprevalence status of PPR in the states of the northern region of India in the sheep and goat populations in the epidemiological units of the studied region. The study suggests that the small ruminants population in majority of the epi-units (n=130) were having a <30% prevalence of PPRV antibodies. This information would be very useful in the formulation of effective disease management strategies as well as to implement the vaccination program. The study also highlights the need for systematic, comprehensive, rigorous, continuous vaccination, and active surveillance programs for few more years to achieve the desired level of PPRV antibodies in the small ruminant populations and to make PPR free zone. Therefore, zoning the PPR risk regions and initiating vaccination program at a specified period with widespread vaccination coverage of all the risk populations in the identified zone or state is of paramount importance for the control and eradication of PPR in India.

## Authors’ Contributions

BV carried out the laboratory test. KVK and GG analyzed the data and edited the manuscript. DM provided diagnostic support. PR provided guidance and support in research work. KPS, DH, and RD designed the sample plan, managed the samples, and prepared GIS map for the study area. VB designed the work with overall monitoring, analyzed the data, drafted, and edited the manuscript. All authors drafted, read, and approved the final manuscript.

## Competing Interests

The authors declare that they have no competing interests.

## Publisher’s Note

Veterinary World remains neutral with regard to jurisdictional claims in the published map and institutional affiliation.

## References

[1] Balamurugan V, Hemadri D, Gajendragad M.R, Singh R.K, Rahman H. (2014). Diagnosis and control of peste des petits ruminants: A comprehensive review. Virusdisease.

[2] Govindaraj G.N, Balamurugan V, Rahman H. (2016). Estimation of economic Loss of PPR in sheep and goats in India:An annual incidence based analysis. Br. J. Virol.

[3] Food and Agriculture Organization (2015). Manual on Global Strategy for the Control and Eradication of Pesti des Petits Ruminants. http://www.fao.org/3/a-i4460e.pdf..

[4] Singh R.P, Saravanan P, Sreenivasa B.P, Singh R.K, Bandyopadhyay S.K. (2004). Prevalence and distribution of peste des petits ruminants virus infection in small ruminants in India. Rev. Sci. Tech.

[5] Singh R.K, Balamurugan V, Bhanuprakash V, Sen A, Saravanan P, Pal Yadav M. (2009). Possible control and eradication of peste des petits ruminants from India: Technical aspects. Vet. Ital.

[6] Govindaraj G.N, Roy G, Mohanty B.S, Balamurugan V, Pandey A.K, Sharma V, Patel A, Mehra M, Pandey S.K, Roy P. (2019). Evaluation of effectiveness of mass vaccination campaign against peste des petits ruminants in Chhattisgarh state, India. Transbound. Emerg. Dis.

[7] Balamurugan V, Govindaraj G.N, Rahman H. (2016). Planning, implementation of peste des petits ruminants control programme and strategies adopted for disease control in India. Br. J. Virol.

[8] Balamurugan V, Muthuchelvan D, Govindaraj G, Roy G, Sharma V, Kumari S.S, Choudhary D, Mohanty B.S, Suresh K.P, Rajak K.K, Hemadri D, Roy P. (2018). Serosurvey for assessing PPR vaccination status in rural system of Chhattisgarh state of India. Small. Rumin. Res.

[9] Balamurugan V, Varghese B, Muthuchelvan D, Kumar K.V, Govindaraj G, Suresh K.P, Kumar P, Hemadri D, Roy P. (2020). Seroprevalence of peste des petits ruminants in sheep and goats in Eastern India. VirusDisease.

[10] Balamurugan V, Varghese B, Muthuchelvan D, Kumari SS, Kumar K.V., Suresh K.P., Govindaraj G, Sunder J, Hemadri D, Roy P. (2019). Cross-sectional seroprevalence study of peste des petits ruminants in goats in Andaman and Nicobar Islands, India. Small Rumin. Res.

[11] Balamurugan V, Varghese B, Muthuchelvan D, Kumari S.S, Kumar K.V, Dheeraj R, Govindaraj G, Suresh K.P, Hemadri D, Roy P. (2020). Seroprevalence of peste des petits ruminants in small ruminants in the North Eastern Region of India. Vet. Ital.

[12] Haq A.A, Santhamani R, Chakravarti S, Yadav A.K, Rajak K.K, Upmanyu V, Sinha D.K, Malik S.Y, Singh R.P. (2017). Investigation on peste des petits ruminants outbreak in goats of Bareilly district of Uttar Pradesh, India. J. Immunol. Immunopathol.

[13] Jindal N, Mahajan N.K, Batra S.M, Mittal D, Khokhar R.S. (2005). Epidemiological observation of peste des petits ruminants in sheep and goat in Haryana. Haryana Vet.

[14] Mahajan V, Filia G, Bal M.S, Kaur G, Sharma S, Dantotia A, Singh C.K. (2017). Outbreaks of peste des petits ruminants (PPR) in Goats in Punjab, India. Int. J. Curr. Microbiol. Appl. Sci.

[15] Singh S, Jindal N, Nain S.P.S, Khokhar R.S. (2006). Seroprevalence of peste des petits ruminants in sheep and goats in and around Haryana state. Haryana Vet.

[16] Balamurugan V, Saravanan P, Sen A, Rajak K.K, Venkatesan G, Krishnamoorthy P, Bhanuprakash V, Singh R.K. (2012). Prevalence of peste des petits ruminants among sheep and goats in India. J. Vet. Sci.

[17] Cochran W.G (1963). Samling Techniques.

[18] Food and Agriculture Organization (2015). Annexe 3.4. post vaccination evaluation tool.

[19] R Core Team (2014). R Core Team, R: A Language and Environment for Statistical Computing R Foundation for Statistical Computing, Vienna, Austria. http://www.R-project.org..

[20] Singh R.P, Sreenivasa B.P, Dhar P, Shah L.C, Bandyopadhyay S.K. (2004). Development of a monoclonal antibody based competitive-ELISA for detection and titration of antibodies to peste des petits ruminants (PPR) virus. Vet. Microbiol.

[21] Thrushfield M (2005). Veterinary Epidemiology.

[22] Snedecor G.W, Cochran W.G. (1967). Statistical Methods.

[23] Acharya N, Poudel S.P, Acharya K.P. (2018). Cross-sectional seroprevalence study of peste des petits ruminants (PPR) in goats of Syangja and Kaski districts of Nepal. Virusdisease.

[24] Al-Majali A.M, Hussain N.O, Amarin N.M, Majok A.A. (2008). Seroprevalence of, and risk factors for, peste des petits ruminants in sheep and goats in Northern Jordan. Prev. Vet. Med.

[25] Almeshay M. D, Gusbi A, Eldaghayes I, Mansouri R, Bengoumi M, Dayhum A. S. (2017). An epidemological study on Peste des petits ruminants in Tripoli Region, Lybia. Vet. Ital.

[26] Dayhum A, Sharif M, Eldaghayes I, Kammon A, Calistri P, Danzetta M.L, Di Sabatino D, Petrini A, Ferrari G, Grazioli S, Pezzoni G, Brocchi E. (2018). Sero-prevalence and epidemiology of peste des petits ruminants in Libya. Transbound. Emerg. Dis.

[27] Faris D, Yilkal A, Berhe G, Kelay B. (2012). Seroprevalence and seroconversion after vaccination against peste des petits ruminants in sheep and goats from Awash Fentale District, Afar, Ethiopia. Prev. Vet. Med.

[28] Swai E.S, Kapaga A, Kivaria F, Tinuga D, Joshua G, Sanka P. (2009). Prevalence and distribution of peste des petits ruminants virus antibodies in various districts of Tanzania. Vet. Res. Commun.

[29] Balamurugan V, Krishnamoorthy P, Raju D.S.N, Rajak K.K, Bhanuprakash V, Pandey A.B, Gajendragad M.R, Prabhudas K, Rahman H. (2014). Prevalence of Peste-des-petits-ruminant virus antibodies in cattle, buffaloes, sheep and goats in India. Virusdisease.

[30] Mahajan S, Agrawal R, Kumar M, Mohan A, Pande N. (2012). Risk of seroconversion to peste des petits ruminants (PPR) and its association with species, sex, age and migration. Small Rumin. Res.

[31] Bhanuprakash V, Saravanan P, Hosamani M, Balamurugan V, Mondal B, Singh R.K. (2008). Status of sheep sera to bluetongue, peste des petits ruminants and sheep pox in a few Northern states of India. Vet. Ital.

[32] Balamurugan V, Saravanan P, Sen A, Rajak K.K, Bhanuprakash V, Krishnamoorthy P, Singh R.K. (2011). Sero-epidemiological study of peste des petits ruminants in sheep and goats in India between 2003 and 2009. Rev. Sci. Tech.

[33] Dixit A.K, Kumar V, Kumar A, Mohan B, Rai B. (2016). Economic losses due to peste des petitis ruminats (PPR) disease in goats:A post outbreak samples study in Auraiya district of Uttar Pradesh. Vet. Pract.

[34] Mahajan S, Agrawal R, Kumar M, Mohan A, Pande N. (2013). Incidence of Peste des petits ruminants in nomadic sheep and goat of Jammu region. Vet. World.

[35] Fournié G, Waret-Szkuta A, Camacho A, Yigezu L.M, Pfeiffer D.U, Roger F. (2018). A dynamic model of transmission and elimination of peste des petits ruminants in Ethiopia. Proc. Natl. Acad. Sci. India.

[36] Zahur A.B, Ullah A, Irshad H, Farooq M.S, Hussain M, Jahangir M. (2009). Epidemiological investigations of a peste des petits ruminants (PPR) outbreak in Afghan sheep in Pakistan. Pak. Vet. J.

